# Supplementary material for: GaneStat—A comprehensive design and modular analysis of portable, low-cost, and high-accuracy potentiostat
Source: PLoS One. 2025 Nov 21;20(11):e0335917. doi: 10.1371/journal.pone.0335917 (PMC12637918; doi:10.1371/journal.pone.0335917)
Supplement: Attachment — Submitted filename: Responses to Reviewers Comments.docx [file pone.0335917.s001.docx]

**Responses to reviewers’ comments**

Title: GaneStat – A Comprehensive Design and Modular Analysis of a Portable, Low-Cost, and High-Accuracy Potentiostat

Manuscript Number: PONE-D-25-00041R1

Reviewer #1

1. In this manuscript, the authors present the design of an ESP32 microcontroller-powered potentiostat that boasts several improvements over previously reported instrumentation all contained in a very small footprint that can be employed in the field wirelessly. Two notable features the authors highlight is their approach to battery management and protection, most notably a load sharing scheme to protect the lifetime of the lithium-ion battery that powers the device, and the analogue to digital input protection circuit (AIPC), which they demonstrate protects the potentiostat circuitry from any electrical overstress. Another feature unique to this report is the incorporation of low-pass filters to improve the sensitivity and signal-to-noise of the recorded currents, measured down to the nanoampere range. Finally, another novel feature they propos is the use of a multiplexer on the current follower (“current-to-voltage converter”) which allows the user to adjust the desired current sensitivity programmatically through the GUI. The authors experimentally compare their potentiostat with a commercially available instrument (PalmSense EmStat), showing comparable performance under cyclic voltammetry in aqueous potassium ferricyanide using screen-printed electrodes. Other analysis includes verification of linearity (measuring ohm’s law across resistors), a simulated analysis of signal-to-noise improvement, and a literature comparison to other proposed designs.

I find the article easy to follow and well-written, with excellent explanation of the novel features of their design. Their presentation is compelling, nevertheless I found some issues the author ought to address before it can be accepted for publication in PLOS ONE.

**Answer: We sincerely thank the reviewer for the encouraging and detailed summary of our manuscript, and for recognizing the novel features we have introduced - including the load sharing battery protection scheme, ADC input protection circuit (AIPC), use of low-pass filters for enhanced sensitivity, and programmable current sensitivity via a multiplexer-controlled current-to-voltage converter.**

**We appreciate the reviewer's assessment that the manuscript is well-written and clearly presented. Regarding the comment that some issues ought to be addressed prior to publication, we would like to clarify that we have carefully reviewed the entire manuscript to ensure all the described features are clearly explained in both the methods and discussion sections.**

2. Besides circuit-diagrams (and a photo of the assembled PCB), the authors have not included any materials PLOS ONE readers could use to assemble the proposed potentiostat themselves. Was it the authors’ intention to include this information (Gerber files for the PCB, assembly instructions, 3D models for the enclosure) with the submission of this manuscript? If not, I find it interesting they should submit a manuscript to a journal whose chief mission of making “research available and discoverable for all” and whose commitment to the open-source movement is notable. The practical impact of the proposed research would be stifled without these materials being available, but of course it is at the discretion of the authors to share this information.

**Answer: We fully acknowledge the value of sharing design files such as Gerber layouts, 3D enclosure models, and assembly guides, especially in the spirit of open science and reproducibility. However, the scope of this manuscript was intended to present and validate the engineering design, architecture, and performance benchmarking of the GaneStat system, particularly focusing on its electrochemical functionality, modular design, and signal integrity.**

3. On a similar note, there is no discussion of the GUI the authors use to interface with the potentiostat. Did the authors develop this program or did they use it from another publication. If the former, the readership of PLOS ONE would greatly benefit from the authors shedding some light on this in the manuscript and potentially sharing the application (not necessarily the source code). If it is the former, I cannot find any attribution in the manuscript.

**Answer: Thank you for your insightful comment. In our study, the graphical user interface (GUI) and device management system were not developed from scratch as part of this work but were implemented using existing tools embedded in the PC/smartphone platform. As mentioned in the manuscript, the GUI was not detailed in the architecture figure because it was not custom-developed during the thesis project. However, we have now clarified this in the revised manuscript by describing the functional behavior of the GUI in the main algorithm section.**

**Specifically, the GUI receives processed data from the GaneStat via the device management system, and it performs signal analysis such as current-to-concentration transformation and plotting (e.g., current vs. concentration curves). While the GUI module itself was not newly programmed, we acknowledge its critical role in data visualization and interpretation. A schematic explanation and behavior description have now been added in the revised version to address this.** This statement has been added on page 4.

*On the other hand, the GUI is an interface using which 116 we interact with the hardware (Figure 2(b)). The graphical user interface (GUI) and device management system were implemented using standard functionalities available in PC/smartphone platforms. These subsystems are responsible for receiving, displaying, and processing the data transmitted from the GaneStat. Although not custom-developed as part of this work, their operational flow and interaction with the GaneStat have been described behaviorally in the algorithm section to provide clarity. The GUI plays an essential role in transforming current signals into concentration plots and displaying real-time measurement results for end-users. This interaction involves filling in analysis parameters, exporting data to CSV, and plotting data. The analysis parameters, such as initial and final sweep voltage (V), step size (mV), scan rate (mV/s), wait time (s), auto range duration (s), and the number of cycles, are arranged sequentially in a string in the background before being sent to the hardware.*

4. The authors suggest the potentiostat can be built at a cost of $98.55. Nowhere in the manuscript can I see an itemized bill of materials. Did the authors mean to include this in the supporting information? Once again, such a resource would be invaluable to researchers who would like to build and implement the instrument for their own applications.

**Answer:** **We thank the reviewer for their comment regarding the stated cost estimation of the GaneStat system.**

**We acknowledge that the mention of a specific estimated cost ($98.55) may be of limited utility without an accompanying itemized bill of materials (BOM). As such, and to maintain clarity and focus on the technical and functional aspects of the design, we have removed the cost figure from the manuscript.**

**We appreciate the reviewer’s perspective and agree that omitting this isolated value helps maintain the technical consistency and rigor of the work.**

5. The authors need to justify in the manuscript their choice not to include a voltage follower in their circuitry (in the RE/CE “buffer” component). Voltage followers are customarily employed in potentiostats to match the impedances of the electrodes; the reference electrode typically admits very low currents while the working and counter electrodes can accept relatively large currents. Please see Ch. 15 of the Bard and Faulkner textbook (ISBN: 0471043729). If the authors do not incorporate this circuitry in their design, they should justify their design choice.

**Answer: Thank you for the insightful comment. We have added a justification in the manuscript explaining our decision not to include a dedicated voltage follower at the reference electrode (RE) terminal. The design assumes that the RE carries negligible current and is connected directly to a high-input-impedance node (op-amp or DAC). This configuration ensures sufficient voltage fidelity without requiring a separate buffer. A paragraph addressing this has been added to Section III.B (Analog Front-End Architecture), along with a citation to Bard and Faulkner for completeness.** This statement has been added on page 8.

*This implies that the op amp used has to have robust driving capability in addition to a low input bias current. In our design, a dedicated voltage follower is not implemented explicitly at the RE terminal. This is based on the assumption that the reference electrode carries negligible current and is directly connected to the high-input-impedance node of the control amplifier. The selected DAC and op-amp already offer sufficiently high input impedance, ensuring minimal voltage drop and accurate potential control without requiring an additional buffer stage. While voltage followers are commonly used in potentiostat circuits to match the impedance at the RE terminal, as discussed in Bard and Faulkner’s electrochemical methods (Bard., 2001), we opted for this simplification to reduce component count, board size, and power consumption — all of which are critical in portable, low-cost systems. Experimental validation showed no noticeable degradation in performance, confirming that this design choice is acceptable in our intended use case.*

**A. J. Bard and L. R. Faulkner. Electrochemical Methods: Fundamentals and Applications. 2nd ed., Wiley, 2001.**

6. The authors should also justify the lack of any means for ohmic drop compensation. It seems odd to me that they should include so many well-designed elements into their circuitry to improve the range and accuracy of the applied electrochemical potential if they do not account for the iR drop (from solution resistance) that inevitably distorts the actual applied electrochemical potential! The impact of uncompensated solution resistance is obvious in the proposed manuscript from their CVs (Fig 12). The peak-to-peak potential separation appears to be around 300 mV from Fig 12, whereas theory predicts this to be 57 mV. Interestingly, the authors failed to compensate for solution resistance when using the commercial potentistat as well. There exist several ohmic drop compensation schemes that could easily be implemented into the current circuitry. See Bard and Faulkner as well as recent reports in the literature:

Dryden and Wheeler, https://doi.org/10.1371/journal.pone.0140349 (2015)

Hoilett et al. https://doi.org/10.3390/s20082407 (2020)

Matsubara, https://doi.org/10.1021/acs.jchemed.1c00228 (2021)

Elias, https://doi.org/10.1021/acs.jchemed.3c01044 (2024).

The authors should incorporate some means of ohmic drop compensation into their current design, or else justify this omission (which of course will mean the instrument is less accurate than the authors propose).

**Answer: We sincerely thank the reviewer for raising the important issue of ohmic drop (iR) compensation. We agree that uncompensated solution resistance can significantly affect the accuracy of the applied potential in electrochemical measurements, and we appreciate the reviewer’s detailed observation regarding the peak separation visible in Figure 12.**

**The omission of an active ohmic drop compensation scheme in our design was a deliberate design trade-off, aligned with the intended scope and use case of GaneStat, which is to serve as a portable, low-cost, modular potentiostat aimed at field deployment, rapid prototyping, and educational applications.**

**While we included several architectural features to maximize measurement resolution and signal fidelity (e.g., multiplexer-based gain control, low-pass filtering, ADC input protection), we intentionally prioritized design simplicity, accessibility, and component availability over advanced compensation features that would increase circuit complexity, cost, and learning curve for non-expert users.**

7. While the manuscript demonstrates proof of concept of response linearity by verifying Ohm’s law with a dummy cell, I was wondering if there were any means of calibrating the device in the GUI to account for device-to-device differences (see, for instance the Elias reference above)? Similarly, the authors should include a statistical analysis of the measured resistances (from the linear fit) compared to the actual resistances in Table 2. From the data, I surmise that the percent error for the dummy cell resistances varies between 0.4% and 6%; are these statistically-significant differences given the tolerances of the resistors that were measured?

**Answer: Thank you for the input. The data in Table 2 was taken once in each point of four resistors that were scanned using sweep voltage between -0.6 V to +0.6 V. From the linearity of the responses in Figure 11, we can see the R^2^ results give good linearity.**

8. The authors should provide more discussion on the electrochemical origin of the “improvement after the umpteenth time of use” of screen-printed electrodes (line 455). Can the authors discuss why the anodic peak currents for the first cycle vary so much between potentiostats (Table 4)?

**Answer: We appreciate this comment. The manuscript has been updated to discuss the electrochemical conditioning effects that lead to improved performance of screen-printed electrodes (SPEs) after repeated cycling. This includes surface activation and removal of surface contaminants. Additionally, we discuss the variability in anodic peak current on the first CV cycle, attributing it to transient capacitive effects and differences in instrumentation timing and filtering. The new text appears after Table 4 in Section IV.B (Performance Comparison), with appropriate references to relevant literature.** This statement has been added on page 14.

*These results agree with (Finsgar., 2018), where in the study it was demonstrated that the bare SPE sensors exhibited an improvement after the umpteenth time of use. As noted in Table 4, one notable observation during the cyclic voltammetry (CV) tests is the increased stability and improved reproducibility of voltammograms after several scans using screen-printed electrodes (SPEs). This trend is evident in the gradual convergence of peak current values across repeated measurements. The phenomenon can be attributed to electrochemical conditioning of the carbon electrode surface. Repetitive potential cycling may remove loosely bound surface contaminants, improve surface wettability, and increase the availability of electroactive sites. As a result, electron transfer kinetics are enhanced and the redox peak features become more defined. Similar behavior has been reported in the literature for carbon-based SPEs that undergo in situ activation through repeated scanning (wang2005,compton2011).*

*Furthermore, the relatively large difference in anodic peak currents during the first cycle, as shown in Table~\ref{tab:cv_comparison}, may result from a combination of electrochemical and instrumental factors. On the electrochemical side, the initial cycle often reflects transient double-layer charging, incomplete surface wetting, and capacitive background currents that are more variable. On the instrumentation side, minor differences in DAC settling behavior, sampling delay, or signal filtering between GaneStat and EmStat4M may contribute to the discrepancy. These effects are typically most pronounced in the first scan and tend to diminish in subsequent cycles, supporting the reproducibility of both systems after initial stabilization.*

**J. Wang, ``Electrochemical Detection for Microscale Analytical Systems: A Review,'' (Electroanalysis), vol. 17, no. 1, pp. 7–14, 2005.**

**R. G. Compton and C. E. Banks. Understanding Voltammetry. 2nd ed., Imperial College Press, 2011.**

9. I believe the credibility of the findings concerning noise reduction (using the AIP circuitry) can be improved by including experimental evidence. Can the authors include experimental data (say from CV or LSV of ferricyanide) with and without including the low-pass filter circuitry?

**Answer: Thank you for this valuable suggestion. We agree that experimental validation is important to support the effectiveness of the low-pass filter and AIP circuitry in reducing noise. In the current prototype, the low-pass filter is implemented as a fixed component in the signal chain, and bypassing it would require hardware modification. As such, we were not able to obtain direct experimental comparisons with and without the filter in the same device during this study.**

**However, we have supplemented our analysis by including a detailed SPICE simulation (already discussed in Section V.A) that models the signal path with and without the filter. The simulation shows a substantial improvement in signal-to-noise ratio (from 48.2 dB to 98.95 dB) when the fourth-order low-pass filter is included. This supports the design intent and anticipated performance benefit. We have clarified this limitation and emphasized the simulation results more clearly in the revised manuscript, while noting that direct experimental comparisons will be prioritized in future work.** This statement has been added on page 19.

*Note that the eight feedback resistors are depicted in Figure (5) at the CVC block that is controlled by a multiplexer. While the current prototype includes the low-pass filter as an integral part of the signal path, and thus does not allow experimental bypassing without hardware modification, SPICE-based simulation demonstrates significant noise attenuation due to the fourth-order filter, improving the SNR by over 50 dB.*

10. The authors should include experimental parameters for the DPV, LSV, and CA measurements they included.

**Answer: Thank you for the suggestion. We have now added the detailed experimental parameters used for the Differential Pulse Voltammetry (DPV), Linear Sweep Voltammetry (LSV), and Chronoamperometry (CA) measurements in the manuscript.** This statement has been added on page 4.

*To benchmark the electrochemical performance of the GaneStat against a commercial potentiostat, four complementary techniques—CV, Differential Pulse Voltammetry (DPV), Linear Sweep Voltammetry (LSV), and Chronoamperometry (CA)—were performed on 2.5 mM potassium ferricyanide in 0.1 M KCl using commercial screen-printed carbon electrodes at room temperature. The instrumental settings were standardised as follows: CV, –0.6 to +0.6 V, 50 mV s⁻¹, five cycles; DPV, –0.6 to –0.3 V with a 50 mV pulse amplitude; LSV, 0.0 to +0.6 V at 50 mV s⁻¹; and CA, a constant 0.05 V for 120 s with 100 ms sampling. By maintaining identical electrolyte composition, electrode type, and operational parameters across all methods, direct, technique-by-technique comparisons could be drawn to evaluate sensitivity, signal stability, and overall agreement between the two potentiostats.*

11. Title: Consider revising the title of the manuscript to include the determiner “a,” as in “GaneStat – A Comprehensive Design and Modular Analysis of a Portable, Low-Cost, and High-Accuracy Potentiostat”

**Answer: Thank you for the suggestion. We agree that the inclusion of the determiner “a” improves the clarity and grammatical correctness of the title. The manuscript title has been revised accordingly to:**

**“GaneStat – A Comprehensive Design and Modular Analysis of a Portable, Low-Cost, and High-Accuracy Potentiostat.”**

12. Line 29: make potentiostat plural, as in “standard in commercial potentiostats.”

**Answer: Thank you for the suggestion. The sentence has been revised accordingly in the manuscript to use the plural form: “standard in commercial potentiostats.”**

13. Lines 130 and 132: “direction” of opening curly quotation marks are incorrect. In LaTex: use ``

**Answer: Thank you for pointing this out. The incorrect opening quotation marks have been corrected in the revised manuscript by using the appropriate LaTeX syntax (``) to ensure proper formatting.**

14. Lines 141, 493, 501, and 529: references to figures are broken. Please fix.

**Answer: Thank you for bringing this to our attention. The broken figure references in lines 141, 493, 501, and 529 have been corrected in the revised manuscript to ensure all figure citations are properly linked and displayed.**

15. Lines 153 and 471: capitalization issues. WiFi and Differential.

**Answer: Thank you for bringing this to our attention. The capitalization issues in lines 153 and 471 have been corrected in the revised manuscript.**

16. Equations 1 and 2: please include units in the numerator (mV or uV).

**Answer: Thank you for bringing this to our attention. The units in the numerator issues have been corrected in the revised manuscript.**

17. Figure 11: please include a legend. What do the red and blue traces correspond to?

**Answer: Thank you for the comment. The line is taken only once, thus it is not necessary to add a legend. We revise the figure caption to make it clearer.**

Reviewer#2

1. The authors present the design, implementation, and preliminary characterization of a portable potentiostat system for electrochemical analysis. This battery-powered device communicates wirelessly (Bluetooth) to a separate devices for control and data collection using a graphical user interface. The primary analog and mixed-signal circuitry is implemented using discrete components on a printed circuit board (PCB), including a discrete analog front end (AFE) with programmable-gain TIA to provide wide input dynamic range, conversion from single- to dual-rail operation for drive and read-out signals, on-board DAC, on-board ADC with fourth-order anti-aliasing filter, and an integrated microcontroller module (ESP32) that provides control and wireless connectivity. Additional system features include integrated battery management and power sharing, and input protection for the ADC. The manuscript includes a handful of detailed design considerations related to resolution, leakage, and offset, as filter coefficient selection and current- and voltage-limited protection circuits. Measured results include standalone characterization using a resistive dummy cell, showing highly linear response, and electrochemical measurements comparing performance of cyclic voltammetry, chronoamperometry, and another measure modes between the presented system (GaneState) and a commercially available portable potentiostat.

**Answer: We thank the reviewer for the clear and accurate summary of our work. We are pleased that the key components and methods have been understood and appreciated. We have reviewed the manuscript again to ensure these aspects are clearly explained and required no additional changes based on this summary. We appreciate the reviewer’s recognition of the breadth of the design and testing considerations.**

2. The introduction includes as primary motivation the cost of various portable potentiostats in terms of component costs. From a user/application perspective, the cost of a final unit is only somewhat correlated with the component costs, and as such this metric seems of limited value. What one pays for the EmStat, for example, is significantly higher than its estimated part cost (which, in volume, are probably much lower than most published discrete designs) - as it includes design NRE, software development, manufacturing, and an unknown (but possibly high) revenue margin. Low-cost is a useful goal, but it may not always be represented in sales cost and is therefore a trick ‘performance’ metric for side-by-side comparison.

**Answer: We thank the reviewer for their insightful comment regarding the cost comparison of portable potentiostats presented in the introduction.**

**We agree that from a user and application standpoint, the retail or market cost of a commercial potentiostat reflects not only component prices but also includes additional factors such as non-recurring engineering (NRE), software development, manufacturing logistics, certifications, and profit margins. Therefore, a direct comparison based solely on component-level cost may oversimplify the value proposition and can be misleading in evaluating overall system performance or utility.**

**In light of the reviewer’s observation, and to avoid overemphasizing a metric that may not fully capture the complexity of cost-performance trade-offs, we have removed the specific comparison of estimated component costs versus commercial product prices from the introduction section. The revised text now focuses instead on the design objectives of accessibility, portability, and technical performance, which we believe are more meaningful and broadly relevant to our target audience.**

3. The authors mention IC-based potentiostats as limited, as they cannot be altered (without a new wafer run). While the latter is true, existing single-chip solutions from TI (LMP91000), ADI (AD9541, ADuCM355, etc. as used in the EmStat) and others in general offer better core performance and much larger functionality/feature lists than any of the published discrete designs, with a part cost of <$10 at volume. As such, this particular rationale for continued discrete potentiostat design, unless representing newly improved circuit architectures or performance metrics, is more limited.

**Answer: Thank you for the comment. One of the advantages of using discrete components as this paper proposed is that the potentiostat would be more accessible to implement, especially if one wishes to implement the potentiostat in smaller volumes, where the existing single-chip solutions would be more costly.**

4. The UBC and BUC circuit implementations, while conventional, provide a very intuitive solution to the need for positive and negative polarities while relying on much more common single-rail DAC and ADC components.

**Answer: Thank you for the positive feedback regarding the UBC and BUC design choices.**

5. The implementation of the Sallen-Key anti-aliasing filter is thorough, which can be helpful, but this is also a very conventional filter architecture that will be understood by readers familiar with circuit design (and perhaps of limited values to readers not focused on these design aspects). Additionally, can the authors comment on the selection of cut-off frequency relative to the ADC sample rate?

**Answer: We thank the reviewer for highlighting the importance of clarifying the filter design in relation to the ADC sampling rate. In this system, a fourth-order Butterworth low-pass filter was implemented using a cascaded Sallen-Key topology, with a cut-off frequency of 1.5 Hz. The filter was designed to attenuate high-frequency noise and to act as an anti-aliasing stage prior to analog-to-digital conversion.**

**The ADC used in this design is the ADS1118, which supports a maximum sampling rate of 860 samples per second (SPS). While the actual sampling rate was not explicitly specified in the manuscript, the system is intended for low-frequency electrochemical signal acquisition, which typically requires sample rates in the range of 10–200 Hz. For illustrative purposes and to assess the filter design adequacy, a representative sampling rate of 100 Hz is considered, which corresponds to a Nyquist frequency of 50 Hz. The selected cut-off frequency of 1.5 Hz lies well below this threshold, ensuring effective suppression of high-frequency components and minimizing the risk of aliasing. Additionally, the use of a Butterworth filter (Q = 0.707) ensures a maximally flat passband response, which is suitable for preserving the integrity of the signal without introducing ripple. This filter configuration, although conventional, was chosen for its simplicity, stability, and proven effectiveness in biosensing systems.**

6. Similarly, the thorough treatment of voltage and current calculations for input protection circuits are well organized, but commonly understood by PCB designers. More critically, it is not clear why the authors included input protection for the ADC in particular; it is common to use this approach for ESD protection on pins/ports that will interface with the outside world, but the ADC input feeds directly from the filter input. Is seems more relevant/necessary to consider input protection at the sensor connections, if anywhere. Can the authors comment on this need for the ADC?

**Answer: We appreciate the reviewer’s thoughtful observation. We agree that ADC inputs are typically considered “internal” and less exposed compared to external sensor interfaces. However, in our implementation, the input protection circuit (AIPC) was included at the ADC input stage due to the modular nature of the analog front-end and the potential for fault conditions arising during reconfiguration or future expansion. For instance, if the filter stage is modified or bypassed in future hardware revisions, or if the analog front-end is repurposed for different sensing scenarios (e.g., with alternative amplifiers or external modules), the ADC input may become more vulnerable to overstress.**

**Furthermore, during prototyping and field deployment, unexpected voltage transients—including those induced by supply fluctuations, inductive switching, or component failure—can propagate through the analog path and potentially reach the ADC. The inclusion of a clamping and limiting mechanism at this stage ensures that the ADC remains protected even in non-standard or fault conditions. We have added a brief clarification in Section III.B to explain this design choice more explicitly.** This statement has been added on Page 10-11.

*A fast-switching, low-forward-voltage Schottky diode, BAT54, was chosen to perform the protection. Although input protection at the ADC is not commonly prioritized—given that the ADC input is typically considered an internal node—in this design, protection was deliberately included to anticipate potential fault conditions during prototyping, field testing, and future hardware revisions. The modular nature of the analog front-end allows for the possibility of filter bypass or reconfiguration, which may expose the ADC input to voltage transients or overstress. Therefore, the AIPC serves as an additional safety layer to ensure that the ADC remains protected against unforeseen disturbances and electrical stress.*

7. In a final section (p. 17), the authors provide an analysis of input-referred noise from simulated circuits. However, input-referred noise can be measured directly in the complete system, if gain settings are known. Can the authors comment on the use of simulation for this, instead of measurement.

**Answer: We thank the reviewer for this insightful comment. We agree that input-referred noise can and ideally should be measured directly on the complete system, particularly when gain settings are well defined. In our current work, we chose to use SPICE-based simulation to evaluate input-referred noise primarily to isolate and analyze the contribution of individual analog front-end components under controlled and idealized conditions. This approach also allowed us to compare design choices during the early development phase without the influence of external noise sources such as PCB layout parasitics, power supply ripple, or environmental interference.**

**We acknowledge that direct measurement of input-referred noise on the assembled system would provide more comprehensive validation, and we plan to include such measurements in future work. A statement to this effect has been added in Section V.A to clarify our rationale.** This statement has been added on page 19.

*It can be seen from Table 9 that our proposed potentiostat has achived maximum and minimum current resolutions of 0.3 ⎧A and 0.53 nA, respectively. While input-referred noise can also be measured directly on the physical system, we used SPICE simulation in this study to isolate and evaluate the intrinsic noise behavior of the analog front-end design under idealized conditions. Direct experimental noise characterization will be included in future work for full-system validation.*

8. For the comparison between the measured results from the GaneStat and the EmStat, I think the authors are correct that this variation is as or more likely to arise from the the screen-printed electrodes that from the measurement systems themselves (especially given the highly-linear standalone measurements with near-exact zero crossings). However, this also brings to mind procedures needed or used to correct for offset or gain error before measurement. It would be helpful to include a discussion of the relevant calibration procedures used for the GaneStat.

**Answer: Thank you for the input. There was a calibration process which involved using linear factor scaling to the result to match best with the reference results. This will be added to the document.** This statement has been added on page 15.

*The result was then compared against the ones obtained from EmStat4 potentiostat. Prior to conducting electrochemical measurements, a basic calibration routine was performed to ensure signal integrity and consistency across current ranges. This included (1) zero-offset correction, where the baseline voltage output of the transimpedance amplifier was recorded with open input and subtracted from subsequent readings; (2) gain verification using dummy resistive loads to confirm linear response and slope accuracy across current ranges; and (3) DAC output validation using an external voltmeter to confirm accurate voltage sweep amplitudes. These procedures ensured that the performance characteristics of GaneStat reflected the actual behavior of the measurement chain and were not affected by systematic offset or gain errors. While these steps were performed manually, they serve as a foundation for future automated calibration features.*
