# Supplementary material for: GaneStat—A comprehensive design and modular analysis of portable, low-cost, and high-accuracy potentiostat
Source: PLoS One. 2025 Nov 21;20(11):e0335917. doi: 10.1371/journal.pone.0335917 (PMC12637918; doi:10.1371/journal.pone.0335917)
Supplement: Attachment — Submitted filename: Response to Reviewers.docx [file pone.0335917.s002.docx]

**Responses to reviewers’ comments**

Title: GaneStat – A Comprehensive Design and Modular Analysis of a Portable, Low-Cost, and High-Accuracy Potentiostat

Manuscript Number: PONE-D-25-00041R1

Reviewer #1

1. Thank you for addressing our issues. While your edits do technically touch on our recommendations, I believe the usefulness of the work—especially in the context of the readership of PLOS ONE—has been diminished in the edits you have made. The authors have seemed to pivot from proposing their GaneStat as an economical alternative to more expensive potentiostats to showcasing some of the design features that readers can implement in their own work. In so doing, the targeted audience are now researchers with good working knowledge of electrochemical instrumentation. Yet, as the authors argue, implementing a simple positive feedback ohmic drop compensation scheme into their design—which would consist of adding a single circuit element, the variable resistor—would “increase circuit complexity, cost, and learning curve for non-expert users.” Who is the target audience here, experts or non-experts? By making compromises to address our comments, the work is in no way improved, and the usefulness of the work is muddied.

**Answer:** We thank the reviewer for their valuable time and insightful feedback. We highly appreciate the reviewer's efforts in assessing the contribution and relevance of our work to the PLOS ONE readership.

We wish to state clearly that the core philosophy and value of GaneStat have not changed: it remains an economical and easily accessible potentiostat alternative.

Our primary target audience is researchers, particularly those working in electrochemistry, materials science, analytical chemistry, and other related disciplines, especially those operating with limited resources.

The decision not to include the Positive Feedback Ohmic Drop Compensation (PFODC) scheme in the basic design represents a trade-off to maintain GaneStat's core value. The fundamental GaneStat design prioritizes simplicity, ease of assembly, and a low learning curve for users of self-made instruments. The addition of an element like a variable resistor for PFODC (while simple for hardware experts) would demonstrably increase the complexity of calibration and troubleshooting for researchers whose focus is on electrochemical applications, not hardware.

In short, GaneStat is an economical and functional alternative that meets the needs of the majority of routine electrochemical experiments.

2. Nevertheless, the article does propose some design features that a much smaller audience may find useful. I suspect such experts are already aware of these, as reviewer #2 mentioned, as many of them seem fairly conventional. Since my expertise is in electrochemistry and not electrical engineering, I cannot speak to the novelty of such design features.

**Answer:** We thank the reviewer for this thoughtful comment. We agree that some of the design features may be considered conventional from an electrical engineering perspective. Our intention was to study, evaluate and improve the potentiostat's typical circuit design and to demonstrate effectively in a low-cost but high resolution analytical instrument for electrochemical research. We believe this practical integration is of value to the electrochemistry community, particularly for laboratories with limited resources.

3. While the manuscript demonstrates proof of concept of response linearity by verifying Ohm’s law with a dummy cell, I was wondering if there were any means of calibrating the device in the GUI to account for device-to-device differences (see, for instance the Elias reference above)? Similarly, the authors should include a statistical analysis of the measured resistances (from the linear fit) compared to the actual resistances in Table 2. From the data, I surmise that the percent error for the dummy cell resistances varies between 0.4% and 6%; are these statistically-significant differences given the tolerances of the resistors that were measured?

**Answer:** Thank you very much for this valuable input. We acknowledge that the resistors used in this work have a tolerance of 5%. Accordingly, we have revised Table 2 to include the resistor values with their ±5% tolerance ranges, together with the measured results.

Regarding calibration, at the present stage our GUI does not yet implement a device-to-device calibration function. As can be seen in Fig. 2b (GUI View in Smartphone), the application primarily displays measurement functions, while calibration is still carried out manually in the programmed device using the regression line shown in Fig. 11. We recognize that integrating a calibration function into the GUI is an important feature for improving measurement accuracy, and it will be incorporated in future works of the system.

In terms of statistical analysis, we compared the measured resistances (from the linear fit) to the actual resistor values. The percent error of the measured resistances lies between 0.36% and 5.83%. For most resistors, the measured error is ≤1%, which is well within both the resistor tolerance and the expected performance range for educational-grade devices (typically 1–5%). The largest deviation (5.83%) occurs for the smallest resistor value (R = 2.7 kΩ), which is consistent with the resistor tolerance and highlights an area for future improvement in our design.

4. I believe the credibility of the findings concerning noise reduction (using the AIP circuitry) can be improved by including experimental evidence. Can the authors include experimental data (say from CV or LSV of ferricyanide) with and without including the low-pass filter circuitry? Experimentally, this amounts to shorting your LPF circuitry, which is easy enough to do.

**Answer:** Thank you for this valuable suggestion. We agree that experimental validation is important to support the effectiveness of the low-pass filter and AIP circuitry in reducing noise. In the current prototype, the low-pass filter is implemented as a fixed component in the signal chain, and bypassing it would require hardware modification. As such, we were not able to obtain direct experimental comparisons with and without the filter in the same device during this study.

However, we have supplemented our analysis by including a detailed SPICE simulation (already discussed in Section V.A) that models the signal path with and without the filter. The simulation shows a substantial improvement in signal-to-noise ratio (from 48.2 dB to 98.95 dB) when the fourth-order low-pass filter is included. This supports the design intent and anticipated performance benefit. We have clarified this limitation and emphasized the simulation results more clearly in the revised manuscript, while noting that direct experimental comparisons will be prioritized in future work.

5. Lines 130 and 132: “direction” of opening curly quotation marks are incorrect. In LaTex: use ``.

**Answer:** We thank the reviewer for pointing this out. We have corrected the quotation marks at lines 130 and 132 by replacing the opening curly quotes with the proper LaTeX syntax.

6. Line 170: capitalization issues. WiFi.

**Answer:** We appreciate the reviewer’s careful observation. The capitalization in Line 170 has been corrected to “WiFi” as suggested.

7. Equations 1 and 2: please include units in the numerator (mV or uV) of the middle expression.

**Answer:** We thank the reviewer for this helpful suggestion. The units (mV) have been added in the numerator of the middle expression in Equations 1 and 2 to ensure clarity and consistency.

8. Figure 11: please include a legend. What do the red and blue traces correspond to?

**Answer:** We appreciate the reviewer’s careful observation. We would like to clarify that Figure 11 actually contains only a single trace. The apparent difference in colour (red and blue) was an artifact from the plotting/exporting process. We have corrected the figure to display a consistent line colour, and no legend is required as there is only one dataset being presented.
